# Nedaplatin-based chemotherapy or cisplatin-based chemotherapy combined with intensity-modulated radiotherapy achieve similar efficacy for stage II-IVa nasopharyngeal carcinoma patients

**DOI:** 10.1038/s41598-022-16216-0

**Published:** 2022-07-13

**Authors:** Chao Deng, Na Zhang, Shun Jiang, Haixia Zhang, Jin’an Ma, Wen Zou, Xianling Liu, Chunhong Hu, Tao Hou

**Affiliations:** 1 Department of Oncology, The Second Xiangya Hospital, Central South University, Changsha, 410012 Hunan People’s Republic of China; 2 Hunan Key Laboratory of Molecular Precision Medicine, Department of Oncology, Xiangya Hospital, Central South University, Changsha, 410011 Hunan People’s Republic of China

**Keywords:** Head and neck cancer, Chemotherapy

## Abstract

This retrospective study compared the efficacy and safety of nedaplatin-based chemoradiotherapy and cisplatin-based chemoradiotherapy in stage II-IVa nasopharyngeal carcinoma (NPC) patients. Patients treated with cisplatin-based or nedaplatin-based chemoradiotherapy between January 2012 and December 2015 were evaluated. Survival was estimated by the Kaplan‒Meier method and compared by the log-rank test. Multivariate analysis was performed using the Cox proportional hazards model. A cohort of 538 NPC patients was enrolled. There were no significant differences in the 5-year overall survival (OS), progression-free survival (PFS), locoregional relapse-free survival (LRRFS), or distant metastasis-free survival (DMFS) between the cisplatin and nedaplatin groups. During the whole treatment course, patients in the cisplatin group had higher incidences of grade 3‒4 vomiting and anorexia, while patients in the nedaplatin group had higher incidences of grade 3‒4 leucopenia and mucositis. In terms of late toxicities, patients in the cisplatin group had a higher incidence of xerostomia. In multivariate analysis, T stage, N stage, and clinical stage were prognostic factors for OS, PFS, and DMFS. In subgroup analyses, nedaplatin-based chemotherapy achieved comparable treatment outcomes in specific populations stratified by age, sex, ECOG PS score and clinical stage. Cisplatin and nedaplatin are effective choices for stage II-IVa NPC patients, with a different spectrum of side effects.

## Introduction

Nasopharyngeal carcinoma (NPC) is the most common head and neck carcinoma in southern China and southeast Asia, and more than 70% of patients are diagnosed at a locally advanced stage (LA-NPC)^1^. Radiotherapy is the major modality of NPC treatment. Intensity-modulated radiotherapy (IMRT) has been the main technique used for NPC patients, achieving a satisfactory local control rate. However, distant metastasis is the major treatment failure pattern in NPC patients, occurring in approximately 30% of patients^2^. Radiotherapy alone achieved satisfactory efficacy in stage I patients, with a 5-year survival rate of approximately 90%^3^. Cisplatin-based concurrent chemoradiotherapy (CCRT) has been established as a standard of care for stage III‒IVa patients^4–6^. However, the merit of chemotherapy in stage II patients remains controversial, particularly in the era of IMRT^7^. Although cisplatin-based chemotherapy benefits the survival of LA-NPC patients, side effects, especially digestive toxicity, nephrotoxicity and ototoxicity, hamper its tolerability and impair patients’ quality of life. It has been reported that the incidence of grade 3/4 toxicity in cisplatin-based CCRT reached 70%^8^. Moreover, cisplatin-based chemotherapy requires hydration during administration to protect renal function, which will prolong the hospital stay. Thus, there is a need for new drugs with similar effectiveness and less toxicity.

Nedaplatin is a second-generation derivative of platinum agents that has a similar anticancer potency and less digestive and renal toxicities than cisplatin^9^. Nedaplatin has been widely used in the treatment of non-small-cell lung cancer^10^, oesophageal carcinoma^11^, and head and neck carcinoma^12^. In the field of NPC treatment, several phase II trials have demonstrated that nedaplatin-based chemotherapy is effective as a second-line and first-line treatment for recurrent and metastatic NPC^13–15^. Nedaplatin is also widely used in the treatment of LA-NPC. Several trials have shown that nedaplatin combined with docetaxel or fluorouracil followed by nedaplatin-based CCRT is an effective and safe regimen for LA-NPC patients^16–18^.

However, reports comparing nedaplatin and cisplatin in LA-NPC patients from the real world with long-term survival outcome results are lacking. In the present study, we retrospectively compared the long-term efficacy and safety of cisplatin-based chemotherapy and nedaplatin-based chemotherapy combined with IMRT.

## Materials and methods

### Patient selection

We retrospectively analysed data from patients with newly diagnosed NPC between January 2012 and December 2015 at the Second Xiangya Hospital, Central South University. All data were extracted from the electronic medical history system of the hospital. The primary tumour site was evaluated by contrast-enhanced magnetic resonance imaging (MRI). Distant metastasis was evaluated by X-ray chest radiography, ultrasonography of the abdominal region and bone scintigraphy or contrast-enhanced computed tomography (CT) of the chest and abdominal region and bone scintigraphy. The inclusion criteria were as follows: (1) pathologically diagnosed nasopharyngeal squamous cell carcinoma; (2) stage II‒IVa according to the 8^th^ edition of the Union for International Cancer Control/American Joint Committee on Cancer (AJCC) staging system; (3) use of induction chemotherapy + CCRT ± adjuvant chemotherapy for stage III‒IVa patients and use of CCRT or radiotherapy ± adjuvant chemotherapy for stage II patients; (4) no previous treatment with radiotherapy, chemotherapy or anti-epidermal growth factor receptor targeted therapy after the diagnosis; and (5) Eastern Cooperative Oncology Group (ECOG) performance status score ≤ 1. The exclusion criteria were as follows: (1) use of different types of platinum drugs in the induction, concurrent, or adjuvant chemotherapy phases; and (2) loss to follow-up within 48 months after treatment started.

The study was approved by the ethics committee of the Second Xiangya Hospital, Central South University, and the requirement to obtain informed patient consent was waived due to the nature of the study. All methods were performed in accordance with the relevant guidelines and regulations.

### Radiotherapy

All patients were treated with IMRT using 6 MV X-rays on Varian 23EX or Varian Trilogy linear accelerators. Each patient was immobilized with a low-temperature thermoplastic film, and simulated positioning was performed on simulated CT. The total dose was as follows: 70–74 Gy/30–33 F to the planning target volume (PTV) of the gross tumour volume (GTVnx) and involved cervical lymph nodes (GTVnd), 60 Gy/2.0 Gy/30 F to the PTV of the high-risk region (CTV1), including the GTVnx and GTVnd, with a margin of 5‒10 mm. As previously reported^18^, the CTV1 region covers the entire nasopharynx, inferior two-thirds of the sphenoid sinus, the anterior third of the clivus, the pterygoid fossae, the posterior third of the nasal cavity and maxillary sinuses, the retropharyngeal nodes, the parapharyngeal space, and the drainage area of the upper neck, and 56 Gy/1.87 Gy/30F was administered to the PTV of the low-risk region (CTV2), including CTV1, plus a margin of 3‒5 mm, the lower neck, and the supraclavicular lymphatic drainage region. All patients received one fraction daily for 5 fractions per week. Dose constraints to adjacent critical organs were applied according to the Radiation Therapy Oncology Group (RTOG) 0225 protocol^19^.

### Chemotherapy

In the cisplatin group, the patients received induction and/or adjuvant chemotherapy with the regimen of docetaxel plus cisplatin (DP; docetaxel 75 mg/m^2^ on day 1 and cisplatin 75 mg/m^2^ d1 or 25 mg/m^2^ on days 1‒3). During radiotherapy, the patients received concurrent cisplatin 100 mg/m^2^ 3-weekly for up to 3 cycles. In the nedaplatin group, the patients received induction and/or adjuvant chemotherapy with the regimen of docetaxel plus nedaplatin (DN; docetaxel 75 mg/m^2^ on day 1 and nedaplatin 75 mg/m^2^ d1 or 25 mg/m^2^ on days 1‒3). During radiotherapy, this group of patients received concurrent nedaplatin 100 mg/m^2^ 3-weekly for up to 3 cycles. In both groups, adjuvant chemotherapy was given to patients who were staged T4 or N3 or those with an inadequate concurrent chemotherapy dose (< 200 mg/m^2^).

Induction chemotherapy and adjuvant chemotherapy were administered every 3 weeks for 1‒3 cycles. In the cisplatin group, 307 (87.7%) patients received a duplex antiemetic prophylaxis regimen (dexamethasone 8 mg iv Qd d1-3 + ondansetron 8 mg iv Bid d1-3), while only 43 (12.3%) patients received a triplex antiemetic prophylaxis regimen (dexamethasone 8 mg iv Qd d1-3 + ondansetron 8 mg iv Qd d1-3 + aprepitant po 125 mg d1, 80 mg d2-3). In the nedaplatin group, 163 (86.7%) patients received duplex antiemetic prophylaxis, and 25 (13.3%) patients received triplex antiemetic prophylaxis.

### Toxicity evaluation and follow-up

Acute toxicities of chemotherapy and radiotherapy were graded during the whole treatment course, including the induction, concomitant, and/or adjuvant phases, according to the National Cancer Institute Common Toxicity Criteria for Adverse Events (CTCAE 5.0) and the Acute and Late Radiation Morbidity Scoring Criteria of the RTOG (Version 3.0)^20^. Acute mucositis and dermatitis were defined as acute inflammation that occurred in the radiation field. Acute haematological toxicities were graded according to routine blood tests during the whole treatment course. Acute non-haematological toxicities, including digestive, liver and renal toxicities, were graded according to the blood test and medical records. Late toxicity, which was defined as toxicity that presented at least 6 months after the end of treatment, was recorded according to the outpatient or telephone follow-up records. All patients were followed up after the completion of the treatment, once every 3 months in the first 2 years, once every 6 months from the 3rd to the 5th year, and once yearly thereafter. Follow-up visits included physical examination, blood biochemistry profile measurement, chest radiography, abdominal ultrasound, endoscopy, and MRI or CT of the head and neck and abdomen as necessary. Patients who did not return for follow-up were contacted via telephone to ascertain their survival status and long-term toxic effects. The last follow-up date was February 1, 2021, and the median follow-up period was 66 months (range: 8‒106 months).

Endpoints included 5-year overall survival (OS), progression-free survival (PFS), locoregional relapse-free survival (LRRFS), and distant metastasis-free survival (DMFS). OS was defined as the interval between the date of treatment initiation and the date of death from any cause or the last follow-up. PFS was defined as the interval between the date of treatment initiation and the date of disease progression, death, or the last follow-up. LRRFS was defined as the interval between the date of treatment initiation and the date of local or regional relapse. DMFS was defined as the interval between the date of treatment initiation and the date of distant metastasis.

### Statistical analysis

Statistical analyses were performed using SPSS V26.0 software (SPSS Inc., Chicago, IL, USA) and R version 3.6.3 (R Foundation for Statistical Computing, https://www.r-project.org/). The characteristics of the patients were compared via the chi-square test or Fisher’s exact test. Survival outcomes, including OS, PFS, LRRFS, and DMFS, were estimated by the Kaplan‒Meier method and compared by the log-rank test and obtained 95% CIs using the Greenwood formula. Multivariate analysis and subgroup analysis of potential prognostic factors was estimated using the Cox proportional hazards model, and an interaction term between treatment methods and the potential prognostic factors was then added into the model to test their interaction effect for survival. In our study, treatment methods and other potential prognostic factors (sex [male or female] and age [< 50 years or ≥ 50 years], ECOG [0 or 1], and cancer stage [II-III or IVA- B]) were entered into the multivariate Cox proportional hazards regression model to test for their main effects, and an interaction term between treatment methods and the potential prognostic factors was then added into the model to test their interaction effect for survival. Two-sided *P* values < 0.05 were statistically significant.

## Results

### Baseline characteristics

There were 724 patients who were treated in our centre between January 2012 and December 2015. Of these patients, 538 patients were enrolled in the present study (Fig. S1). Among all the patients meeting the inclusion criteria, 54 patients were excluded because of inadequate follow-up. The patient and disease characteristics are listed in Table 1. Among all the patients, 189 (35.1%) patients were older than 50 years, 377 (70.1%) were male, and 292 (54.3%) were smokers. A total of 489 (90.9%) patients had an ECOG PS score of 0. According to the AJCC 8th edition staging system, the patients enrolled in the present study were divided into stage II (12.8%), stage III (61.5%), and stage IVa (25.7%). There were no statistically significant differences in the proportional distributions of age, sex, T stage, N stage, or clinical stage between the two groups. Of the 69 stage II patients enrolled, 28 received CCRT. Therefore, 497 (92.4%) patients in the whole cohort received CCRT. Of these patients, 342 (63.6%) patients had a dose reduction during CCRT, which was defined as receiving a total dose of cisplatin or nedaplatin < 200 mg/m^2^. The proportion of patients with dose adjustment was higher in the cisplatin group than in the nedaplatin group (67.1% vs. 56.9%, *P* = 0.024). In the cisplatin group, the dose reduction was primarily due to severe mucositis and its induced malnutrition (115/235, 48.9%) and bone marrow suppression (102/235, 43.4%). In the nedaplatin group, the dose reduction was mostly due to bone marrow suppression (61/107, 57%), followed by mucositis and malnutrition (35/107, 32.7%). A total of 477 (88.7%) patients received induction chemotherapy, while 462 (85.9%) patients received at least one cycle of adjuvant chemotherapy.

**Table 1:** Baseline characteristics of the patients.

Characteristics	Cisplatin (*n* = 350)	Nedaplatin (*n* = 188)	*P* value
**Age**			
< 50 years	235 (67.1%)	114 (60.6%)	0.155
≥ 50 years	115 (32.9%)	74 (39.4%)	
**Sex**			
Male	254 (72.6%)	123 (65.4%)	0.093
Female	96 (27.4%)	65 (34.6%)	
**Smoking status**			
Non/ex-smoker	159 (45.4%)	87 (46.3%)	0.856
smoker	191 (54.6%)	101 (53.7%)	
**ECOG PS**			
0	320 (91.4%)	169 (89.9%)	0.638
1	30 (8.6%)	19 (10.1%)	
**T stage**			
1	46 (13.1%)	19 (10.1%)	0.150
2	124 (35.4%)	83 (44.1%)	
3	122 (34.9%)	64 (34.0%)	
4	58 (16.6%)	22 (11.7%)	
**N stage**			
0	17 (4.9%)	13 (6.9%)	0.706
1	63 (18.0%)	31 (16.5%)	
2	227 (64.9%)	118 (62.8%)	
3	43 (12.3%)	26 (13.8%)	
**Clinical stage**			
II	45 (12.9%)	24 (12.8%)	0.536
III	210 (60.0%)	121 (64.4%)	
IVa	95 (27.1%)	43 (22.9%)	
**Dose reduction**			
Yes	235 (67.1%)	107 (56.9%)	**0.024**
No	115 (32.9%)	81 (43.1%)	
**Induction chemotherapy**			
0	40 (11.4%)	21 (11.1%)	
1	273 (78.0%)	109 (58.0%)	
2	25 (7.2%)	40 (21.3%)	
3	12 (3.4%)	13 (6.9%)	
4	0 (0.0%)	5 (2.7%)	
**Adjuvant Chemotherapy**			
0	47(13.4%)	29 (15.4%)	
1	70 (20.0%)	39 (20.7%)	
2	83 (23.7%)	65 (34.6%)	
3	147 (42.0%)	49 (26.1%)	
4	2 (0.6%)	6 (3.2%)	
5	1 (0.3%)	0 (0.0%)	

Significant values are in [bold].

*PS* Eastern Cooperative Oncology Group Performance Status.

### Survival outcomes

**Figure 1:**
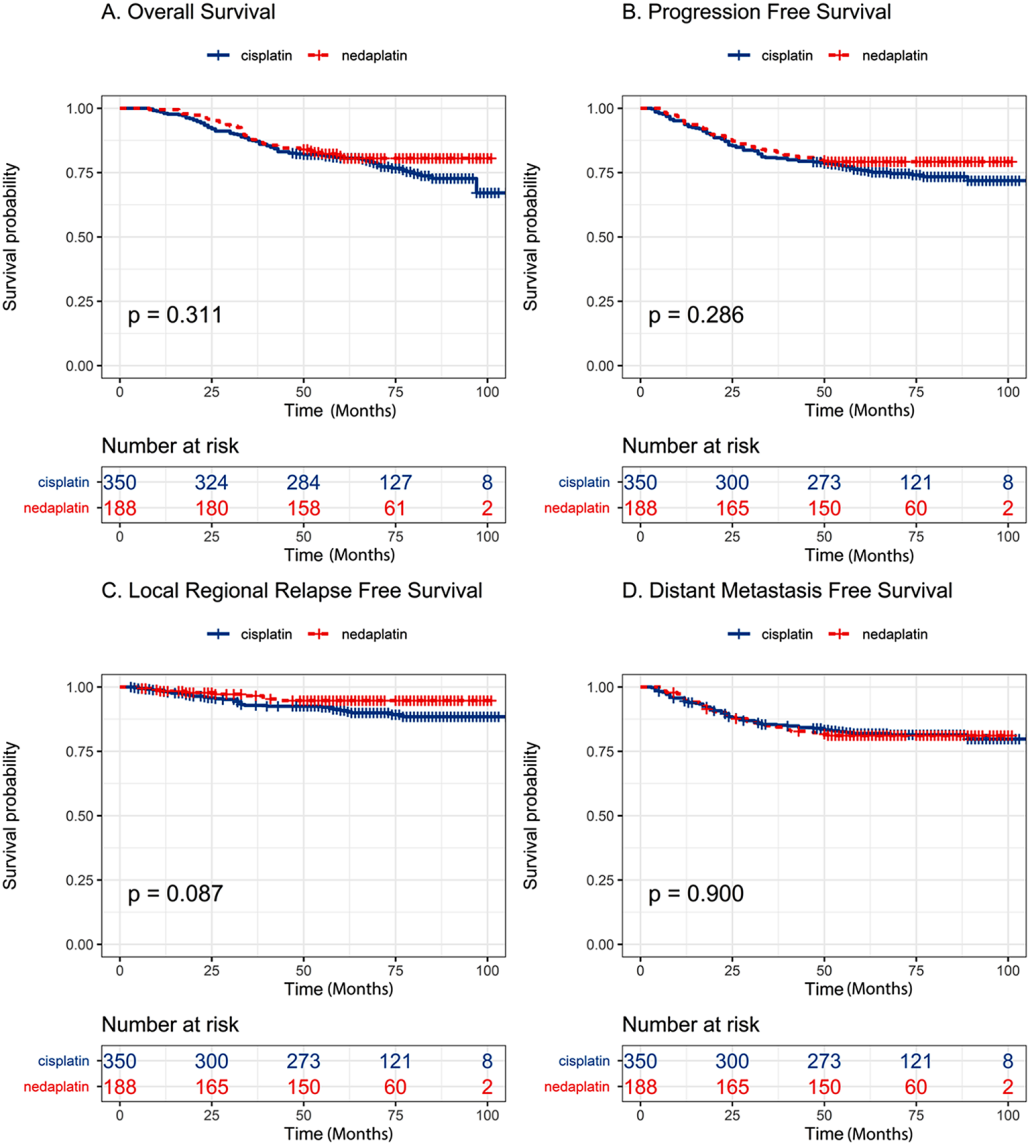
Kaplan‒Meier survival curves for radiotherapy with cisplatin-based chemotherapy or nedaplatin-based chemotherapy in the 538 patients with stage II‒IVa nasopharyngeal carcinoma. (**A**) Overall survival. (**B**) Progression-free survival. (**C**) Locoregional relapse-free survival. (**D**) Distant metastasis-free survival. P values were calculated using the log-rank test.

The median follow-up period was 66 months (ranging from 8 to 106 months). As shown in Fig. 1 and Table 2, the 5-year OS rates were 80.6% (95% CI, 76.6–84.9%) in the cisplatin group and 81.5% (95% CI, 76.0–87.3%) in the nedaplatin group (HR, 0.815; 95% CI, 0.548–1.212; log-rank P = 0.311). The 5-year PFS rates were 75.9% (95% CI, 71.5–80.0%) in the cisplatin group and 79.2% (95% CI, 73.6%-85.3%) in the nedaplatin group (HR, 0.8150; 95% CI, 0.559–1.188; log-rank P = 0.286). The 5-year LRRFS rates were 90.9% (95% CI, 87.7–94.2%) in the cisplatin group and 94.7% (95% CI, 91.4–98.2%) in the nedaplatin group (HR, 0.530; 95% CI, 0.253–1.110; log-rank *P* = 0.087). The 5-year DMFS rates were 82.3% (95% CI, 78.4–86.5%) in the cisplatin group and 81.2% (95% CI, 75.7–87.0%) in the nedaplatin group (HR, 1.027; 95% CI, 0.679–1.552; log-rank *P* = 0.900).

**Table 2:** Univariate analysis of potential factors associated with survival.

Variables	5-y OS		5-y PFS		5-y LRRFS		5-y DMFS	
	%	*P*	%	*P*	%	*P*	%	*P*
**Age**								
≥ 50 years	80.2	0.990	76.3	0.552	92.6	0.823	82.0	0.938
< 50 years	82.3		77.4		92.0		81.5	
**Sex**								
Male	79.5	0.267	76.2	0.418	92.5	0.728	80.4	0.214
Female	84.4		79.1		91.5		84.6	
**ECOG PS**								
0	81.6	0.128	78.1	0.079	91.8	0.375	83.0	**0.026**
1	74.8		66.4		96.3		68.9	
**Smoking status**								
Non/ex-smoker	83.5	0.241	78.0	0.438	92.5	0.679	82.9	0.391
Smoker	78.8		76.2		92.0		80.6	
**T stage**								
1‒2	85.7	**0.001**	82.3	**0.001**	94.8	**0.006**	85.2	**0.021**
3‒4	76.0		71.6		89.6		78.0	
**N stage**								
0‒1	92.5	** < 0.001**	87.8	**0.001**	94.8	0.237	92.6	** < 0.001**
2‒3	77.5		73.8		91.3		78.4	
**Clinical stage**								
II‒III	85.8	** < 0.001**	81.9	** < 0.001**	92.7	0.144	86.7	** < 0.001**
Iva	66.8		62.8		90.8		67.1	
**Drug type**								
Cisplatin	80.6	0.311	75.9	0.286	90.9	0.087	82.0	0.900
Nedaplatin	81.5		79.2		94.7		81.2	

Significant values are in [bold].

*OS* overall survival, *PFS* progression-free survival, *LRRFS* locoregional relapse-free survival, *DMFS* distant metastasis-free survival, *ECOG PS* Eastern Cooperative Oncology Group Performance Status.

### Subgroup analysis

We performed subgroup analyses for OS, PFS, DMFS, and LRRFS in patients stratified by the following variates: age (< 50 or ≥ 50), sex (male or female), ECOG PS score (0 or 1), and disease stage (II-III or IVa). No interactions between these variates and treatment outcome were observed (Fig. S2), suggesting that nedaplatin-based chemotherapy could achieve comparable treatment outcomes among specific populations.

### Prognostic factors

To determine the factors affecting patient survival, we performed univariate and multivariate analyses to identify the factors predicting OS, PFS, LRRFS, and DMFS. The putative prognostic factors included sex, age, ECOG PS, smoking history, T stage, N stage, clinical stage, and drug type (Table 2). In univariate analysis, we found that T stage, N stage, and clinical stage were prognostic factors for OS, PFS, and DMFS. T stage was also a prognostic factor for LRRFS. ECOG PS was a prognostic factor for DMFS.

**Figure 2:**
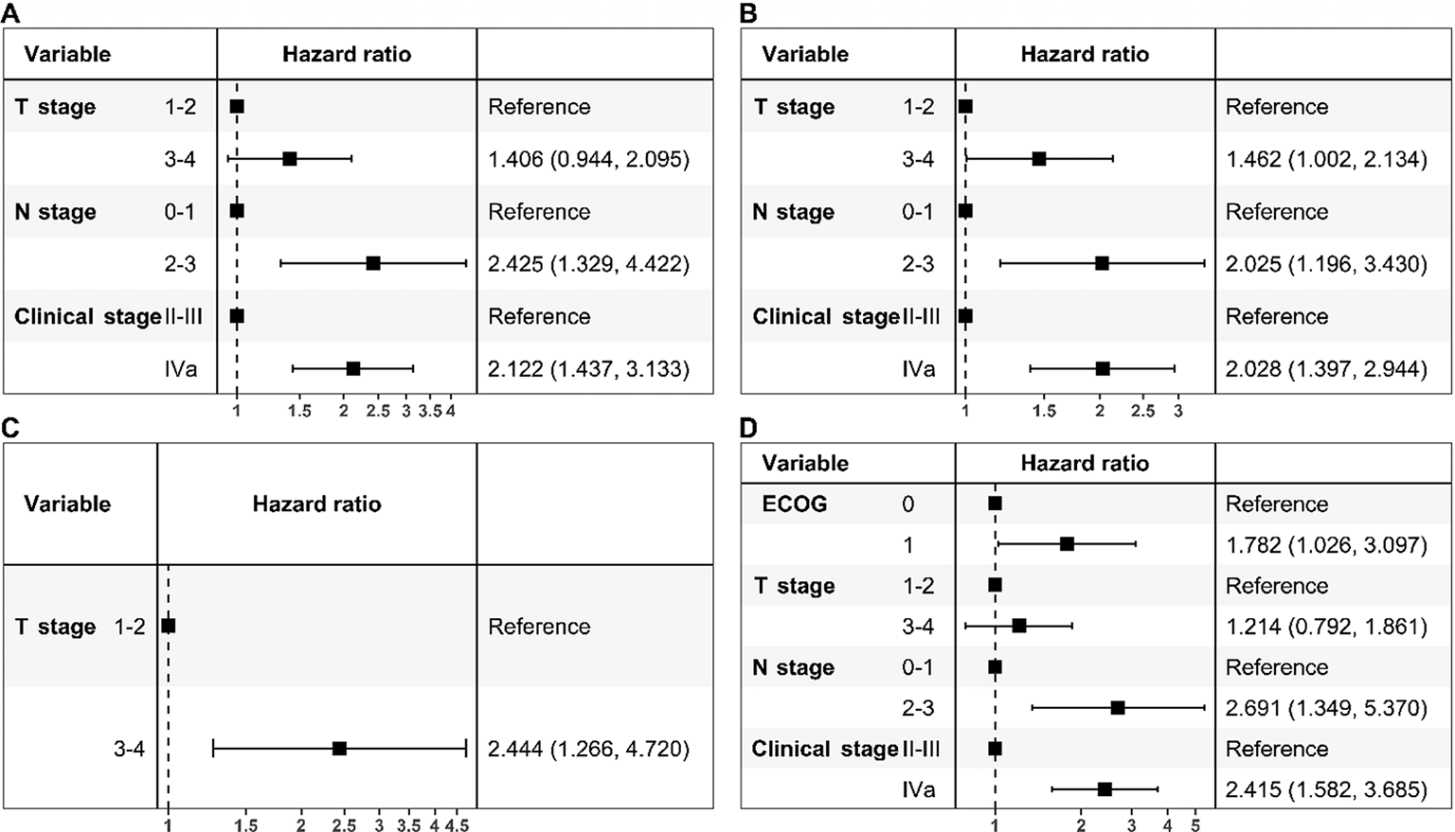
Forest plot based on multivariate analysis-based adjusted hazard ratios (HRs) from Cox regression for overall survival (**A**), progression-free survival (**B**), locoregional relapse-free survival (**C**), and distant metastasis-free survival (**D**) in the entire cohort (n = 538).

Further multivariate analyses showed that clinical stage and N stage were independent predictive factors of OS, PFS, and DMFS. T stage was an independent predictive factor of PFS and LRRFS. Moreover, ECOG PS was an independent predictor of DMFS (Table 3, Fig. 2).

**Table 3:** Multivariate analysis of factors potentially associated with survival.

Variables	5-y OS		5-y PFS		5-y LRRFS		5-y DMFS	
	*P* value	HR(95% CI)	*P* value	HR(95% CI)	*P* value	HR(95% CI)	*P* value	HR(95% CI)
**ECOG PS**								
0							***0.040***	1.782(1.026–3.097)
1								
**T stage**								
1–2	0.094	1.406(0.944–2.095)	***0.049***	1.462(1.002–2.134)	***0.001***	1.856(1.297–2.656)	0.374	1.214(0.792–1.861)
3–4								
**N stage**								
0–1	***0.004***	2.425(1.329–4.422)	***0.009***	2.025(1.196–3.430)			***0.005***	2.691(1.349–5.370)
2–3								
**Clinical stage**								
II-III	** < *0.001***	2.122(1.437–3.133)	** < *0.001***	2.028(1.397–2.944)			** < *0.001***	2.415(1.582–3.685)
IVa								

Significant values are in [bolditalic].

*OS* overall survival, *PFS* progression-free survival, *LRRFS* locoregional relapse-free survival, *DMFS* distant metastasis-free survival, *ECOG PS* Eastern Cooperative Oncology Group Performance Status, *HR* hazard ratio.

### Toxicity

The treatment-related acute and late toxicities are listed in Table 4. Generally, patients in the cisplatin group had a higher incidence of non-haematological toxicities, while patients in the nedaplatin group had a higher incidence of haematological toxicities. The proportions of patients with grade ≥ 3 leucopenia and grade ≥ 3 neutropenia in the nedaplatin group were significantly higher than those in the cisplatin group (*P* = 0.015 and *P* = 0.007, respectively). The proportions of patients with grade ≥ 1 nausea, grade ≥ 1 vomiting, grade ≥ 3 vomiting, and grade ≥ 1 and grade ≥ 3 anorexia in the cisplatin group were significantly greater than those in the nedaplatin group (*P* < 0.001, *P* < 0.001, *P* = 0.002, *P* = 0.007, and *P* < 0.001, respectively). Although patients in the cisplatin group had a higher incidence of constipation (*P* = 0.024), the incidence of severe constipation was not significantly different between the two groups. The proportion of patients with grade ≥ 3 mucositis was significantly higher in the nedaplatin group than in the cisplatin group (*P* = 0.018). However, the incidences of acute kidney injury and cardiotoxicity were similar between the two groups. In terms of late toxicities, the incidences of ototoxicity and dysphagia were not significantly different between the two groups. However, patients in the cisplatin group had a higher incidence of xerostomia (*P* = 0.036).

**Table 4:** Frequency of acute and late toxicities in the two groups.

	Cisplatin			Nedaplatin			*P* value for events grade ≥ 1	*P* value for events grade ≥ 3
	Any Grade	Grade 1–2(%)	Grade 3–4(%)	Any Grade	Grade 1–2(%)	Grade 3–4(%)		
**Haematological**								
Leucopenia	252(72.0)	168(48.0)	84(24.0)	148(78.7)	84(44.7)	64(34.0)	0.098	***0.015***
Neutropenia	216(61.7)	193(55.1)	23(6.6)	128(68.1)	102(54.3)	26(13.8)	0.158	***0.007***
Anaemia	217(62.0)	217(62.0%)	0(0)	132(70.2)	131(69.7)	1(0.5)	0.059	0.349
Thrombocytopenia	67(19.2)	66(18.9)	1(0.3)	41(21.8)	38(20.2)	3(1.6)	0.499	0.125
**Non-haematological**								
Nausea	310(88.6)	309(88.3)	1(0.3)	139(73.9)	139(73.9)	0(0)	** < *0.001***	1.000
Vomiting	267(76.3)	243(69.4)	24(6.9)	41(21.8)	39(20.7)	2(1.1)	** < *0.001***	***0.002***
Anorexia	196(56.0)	144(41.1)	52(14.9)	82(43.6)	75(39.9)	7(3.7)	***0.007***	** < *0.001***
Constipation	138(39.5)	136(38.9)	2(0.6)	54(28.7)	53(28.2)	1(0.5)	***0.024***	1.000
Hypoalbuminemia	158(45.1)	158(45.1)	0(0)	77(41.0)	77(41.0)	0(0)	0.141	NA
Transaminase increase	69(19.7)	69(19.7)	0(0)	34(18.1)	34(18.1)	0(0)	0.730	NA
Creatinine increase	31(8.9)	31(8.9)	0(0)	8(4.3)	8(4.3)	0(0)	0.055	NA
Mucositis	343(98.0)	273(78.0)	70(20.0)	180(95.8)	125(66.5)	55(29.3)	0.169	***0.018***
Dermatitis	322(92.0)	299(85.4)	23(6.6)	180(95.8)	171(91.0)	9(4.8)	0.077	0.451
**Late toxicities**								
Ototoxicity	149(43.6)	NA	NA	76(40.6)	NA	NA	0.521	NA
Xerostomia	196(57.3)	NA	NA	89(47.6)	NA	NA	***0.036***	NA
Dysphagia	52(15.2)	NA	NA	25(13.4)	NA	NA	0.608	NA

Significant values are in [bolditalic].

## Discussion

In the present study, we retrospectively analysed the treatment of LA-NPC patients with cisplatin- or nedaplatin-based chemotherapy combined with IMRT. Patients in the cisplatin and nedaplatin groups had similar survival rates. The differences in the 5-year OS, PFS, LRRFS, and DMFS rates between the two groups were not statistically significant. In terms of acute toxicity, there were different spectra of acute toxicities between the two groups. The incidence of haematological toxicities was significantly higher in the nedaplatin group, while that of gastrointestinal tract toxicities was significantly higher in the cisplatin group. Moreover, the incidence of xerostomia was higher in the cisplatin group. In multivariate analysis, we found that T stage, N stage, clinical stage, and ECOG PS were major prognostic factors, which was in accordance with previous reports^21–23^.

Cisplatin-based chemotherapy is the regimen most widely used in the treatment of metastatic^24^ and locoregional advanced^6^ NPC. The 5-year OS rate of stage III‒IV patients after IC + CCRT was approximately 80.8%^25^. Some trials have reported that CCRT brings survival benefits to stage II patients, with a 5-year OS rate of 94.5%^26^. Although cisplatin-based chemotherapy brought benefits to NPC patients, it also resulted in increased toxicities, which decreased its tolerability and the quality of life of the patients. Nedaplatin is a second-generation platinum derivative with lower rates of renal and gastrointestinal toxicities than cisplatin. Due to its lower renal toxicity, nedaplatin does not require hydration in clinical use.

During the past decade, there has been increasing evidence showing that nedaplatin is as effective as cisplatin in the treatment of NPC. In a retrospective study, Liu et al.^27^ reported that nedaplatin combined with fluorouracil, followed by nedaplatin concurrent with IMRT, achieved 5-year OS, PFS, LRFS, and DMFS rates of 82.4%, 86.2%, 91.0%, and 80.7%, respectively, which were similar to those with cisplatin-based chemotherapy. In a prospective phase III trial, Tang et al.^18^ made a head-to-head comparison of cisplatin and nedaplatin in stage II-IVa NPC patients. They reported that the 2-year PFS rate in the nedaplatin group was 88.0%, which was comparable to that in the cisplatin group. Later, they updated the results and reported that the primary endpoint, the 5-year PFS rate, was not significantly different between the nedaplatin group and the cisplatin group (81.4% vs. 79.8%). Moreover, no significant survival differences were observed in the 5-year OS (89.4% vs. 88.8%, *P* = 0.63), DMFS (85.9% vs. 90.4%, *P* = 0.17), or LRRFS (92.6% vs. 89.6%, *P* = 0.17) rates between the two groups^28^. In our study, we found that the 5-year PFS rates in the nedaplatin and cisplatin groups were 79.2% and 75.9%, the 5-year OS rates were 81.5% and 80.6%, and the 5-year DMFS rates were 82% and 81.2%, respectively, which were lower than the data from clinical trials. However, the 5-year LRRFS was comparable to that in the clinical trial. This result indicated that the patients in our cohort had a higher incidence of distant metastasis. This may result from a higher proportion of concurrent drug dose reductions. In the present study, 63.6% of the patients received a concurrent drug dose of < 200 mg/m^2^. Ng WT et al. reported that patients who received less than 2 cycles of concurrent cisplatin (200 mg/m^2^) had inferior disease control^29^. Moreover, there are about 10% of patients who were excluded due to inadequate follow-up. Considering that the risk of disease relapse is very low for those patients who had no disease relapse at 48 months after the treatment, this group of patients may cause underestimation the actual survival rate in the whole cohort.

In terms of toxicities, it was reported that the overall percentage of grade ≥ 3 adverse events was 73%, and approximately two-thirds of the patients discontinued concurrent cisplatin treatment^8^. In a retrospective study, Liu et al.^27^ reported that the nedaplatin group had higher incidences of grade 3‒4 neutropenia and thrombocytopenia, while the cisplatin group had higher incidences of grade 3‒4 nausea, vomiting, and weight loss. It was reported in a prospective study that the incidence of grade 3‒4 nausea and vomiting was significantly higher in the cisplatin group, although the incidence of haematological events was similar between the two groups^18^. Later, they reported in the secondary analysis that patients in the cisplatin group had a higher incidence of grade 3 and 4 auditory toxic effects than those in the nedaplatin group^28^. In the present study, we found that the frequency of grade 3 or 4 leucopenia was 27.51% (148/538), while it was significantly higher in the nedaplatin group. Our results indicated that the surveillance of haematological toxicities should be more emphasized in patients treated with nedaplatin-based chemotherapy in the real world. The frequency of grade 3 or 4 vomiting in the cisplatin group was 6.9%, which was also lower than previously reported rates. This may be attributed to splitting of the cisplatin dose. However, the incidence of hypoalbuminemia induced by vomiting and mucositis between the two groups had no statistical significance. In the present study, the incidence of acute renal toxicity was higher in the cisplatin group, with borderline statistical significance, which may be due to splitting of the cisplatin dose and adequate hydration before treatment. Regarding late toxicities, we found that the incidence of xerostomia was significantly higher in the nedaplatin group, and the incidence of ototoxicity was similar between the two groups. However, due to the limitation of follow-up data, we could not grade the severity of the toxicities.

Certainly, there were a few limitations to the study. First, the retrospective nature and long time span of the study may have introduced bias to the results. Second, the proportion of patients who received a dose adjustment in the nedaplatin group was higher than that in the cisplatin group, which may be a confounding factor. Third, the incidence and severity of some long-term toxicities, such as neurotoxicity, were not reported in the present study. Fourth, there were some clinical characteristics that play an important role in prognosis and treatment optimization, such as Epstein‒Barr virus DNA and lactate dehydrogenase levels^30^, that were not taken into consideration. Finally, the results should be interpreted with caution when applying them to patients from nonendemic regions, such as Europe and America.

In conclusion, our study demonstrated that cisplatin-based chemotherapy and nedaplatin-based chemotherapy achieved comparable survival, with different toxicity profiles, in patients with NPC. Nedaplatin may be an alternative choice for NPC patients, particularly for those who are at high risk of severe gastrointestinal toxicities or who cannot tolerate cisplatin due to kidney morbidity.

## Supplementary Information

Supplementary Information 1.

Supplementary Information 2.

## Data Availability

The datasets used and/or analysed during the current study are available from the corresponding author on reasonable request.

## References

[1] Chua MLK, Wee JTS, Hui EP, Chan ATC (2016). Nasopharyngeal carcinoma. Lancet.

[2] Chen L (2019). 10-Year results of therapeutic ratio by intensity-modulated radiotherapy versus two-dimensional radiotherapy in patients with nasopharyngeal carcinoma. Oncologist.

[3] Yi JL (2006). Nasopharyngeal carcinoma treated by radical radiotherapy alone: Ten-year experience of a single institution. Int. J. Radiat. Oncol. Biol. Phys..

[4] Chen L (2012). Concurrent chemoradiotherapy plus adjuvant chemotherapy versus concurrent chemoradiotherapy alone in patients with locoregionally advanced nasopharyngeal carcinoma: a phase 3 multicentre randomised controlled trial. Lancet Oncol..

[5] Li XY (2019). Ten-year outcomes of survival and toxicity for a phase III randomised trial of concurrent chemoradiotherapy versus radiotherapy alone in stage II nasopharyngeal carcinoma. Eur. J. Cancer.

[6] Zhang Y (2019). Gemcitabine and cisplatin induction chemotherapy in nasopharyngeal carcinoma. N. Engl. J. Med..

[7] Liu DH (2020). Survival of stage II nasopharyngeal carcinoma patients with or without concurrent chemotherapy: A propensity score matching study. Cancer Med..

[8] Sun Y (2016). Induction chemotherapy plus concurrent chemoradiotherapy versus concurrent chemoradiotherapy alone in locoregionally advanced nasopharyngeal carcinoma: a phase 3, multicentre, randomised controlled trial. Lancet Oncol..

[9] Shimada M, Itamochi H, Kigawa J (2013). Nedaplatin: a cisplatin derivative in cancer chemotherapy. Cancer Manag. Res..

[10] Shukuya T (2015). Nedaplatin plus docetaxel versus cisplatin plus docetaxel for advanced or relapsed squamous cell carcinoma of the lung (WJOG5208L): a randomised, open-label, phase 3 trial. Lancet Oncol..

[11] Ueda H (2019). Phase II trial of 5-fluorouracil, docetaxel, and nedaplatin (UDON) combination therapy for recurrent or metastatic esophageal cancer. Oncologist.

[12] Wei XF (2020). Neoadjuvant chemotherapy as a comprehensive treatment in patients with laryngeal and hypopharyngeal carcinoma. Acta Otolaryngol..

[13] Peng PJ (2017). Multi-institutional prospective study of nedaplatin plus S-1 chemotherapy in recurrent and metastatic nasopharyngeal carcinoma patients after failure of platinum-containing regimens. Ther. Adv. Med. Oncol..

[14] Peng PJ (2015). Phase II trial of docetaxel combined with nedaplatin for patients with recurrent and metastatic nasopharyngeal carcinoma. Drug Des. Dev. Ther..

[15] Peng PJ (2013). Multicenter phase II study of capecitabine combined with nedaplatin for recurrent and metastatic nasopharyngeal carcinoma patients after failure of cisplatin-based chemotherapy. Cancer Chemother. Pharmacol..

[16] Tang C (2016). Comparison between nedaplatin and cisplatin plus docetaxel combined with intensity-modulated radiotherapy for locoregionally advanced nasopharyngeal carcinoma: a multicenter randomized phase II clinical trial. Am. J. Cancer Res..

[17] Zheng J (2010). Induction chemotherapy with nedaplatin with 5-FU followed by intensity-modulated radiotherapy concurrent with chemotherapy for locoregionally advanced nasopharyngeal carcinoma. Jpn. J. Clin. Oncol..

[18] Tang LQ (2018). Concurrent chemoradiotherapy with nedaplatin versus cisplatin in stage II-IVB nasopharyngeal carcinoma: an open-label, non-inferiority, randomised phase 3 trial. Lancet Oncol..

[19] Lee N (2009). Intensity-modulated radiation therapy with or without chemotherapy for nasopharyngeal carcinoma: radiation therapy oncology group phase II trial 0225. J. Clin. Oncol..

[20] Cox JD, Stetz J, Pajak TF (1995). Toxicity criteria of the radiation therapy oncology group (RTOG) and the european organization for research and treatment of cancer (EORTC). Int. J. Radiat. Oncol. Biol. Phys..

[21] Li QJ (2020). A nomogram based on serum biomarkers and clinical characteristics to predict survival in patients with non-metastatic nasopharyngeal carcinoma. Front Oncol..

[22] Duan YY (2021). Construction of a comprehensive nutritional index and comparison of its prognostic performance with the PNI and NRI for survival in older patients with nasopharyngeal carcinoma: a retrospective study. Support Care Cancer.

[23] Lai C (2021). A novel prognostic model predicts overall survival in patients with nasopharyngeal carcinoma based on clinical features and blood biomarkers. Cancer Med..

[24] Zhang L (2016). Gemcitabine plus cisplatin versus fluorouracil plus cisplatin in recurrent or metastatic nasopharyngeal carcinoma: a multicentre, randomised, open-label, phase 3 trial. Lancet.

[25] Yang Q (2019). Induction chemotherapy followed by concurrent chemoradiotherapy versus concurrent chemoradiotherapy alone in locoregionally advanced nasopharyngeal carcinoma: long-term results of a phase III multicentre randomised controlled trial. Eur. J. Cancer.

[26] Chen QY (2011). Concurrent chemoradiotherapy vs radiotherapy alone in stage II nasopharyngeal carcinoma: phase III randomized trial. J. Natl. Cancer Inst..

[27] Liu T (2018). Neoadjuvant chemotherapy with fluorouracil plus nedaplatin or cisplatin for locally advanced nasopharyngeal carcinoma: a retrospective study. J. Cancer.

[28] Tang QN (2021). Effect of concurrent chemoradiotherapy with nedaplatin vs cisplatin on the long-term outcomes of survival and toxic effects among patients with stage II to IVB nasopharyngeal carcinoma: a 5-year follow-up secondary analysis of a randomized clinical trial. JAMA Netw. Open.

[29] Ng WT (2018). Concurrent-adjuvant chemoradiation therapy for stage III-IVB nasopharyngeal carcinoma-exploration for achieving optimal 10-year therapeutic ratio. Int. J. Radiat. Oncol. Biol. Phys..

[30] Tang SQ (2020). Induction versus adjuvant chemotherapy combined with concurrent chemoradiotherapy in locoregionally advanced nasopharyngeal carcinoma: A propensity score-matched analysis. Oral Oncol..

