# Supplementary figures and images for: Nedaplatin-based chemotherapy or cisplatin-based chemotherapy combined with intensity-modulated radiotherapy achieve similar efficacy for stage II-IVa nasopharyngeal carcinoma patients
Source: Sci Rep. 2022 Jul 13;12:11978. doi: 10.1038/s41598-022-16216-0 (PMC9279476; doi:10.1038/s41598-022-16216-0)

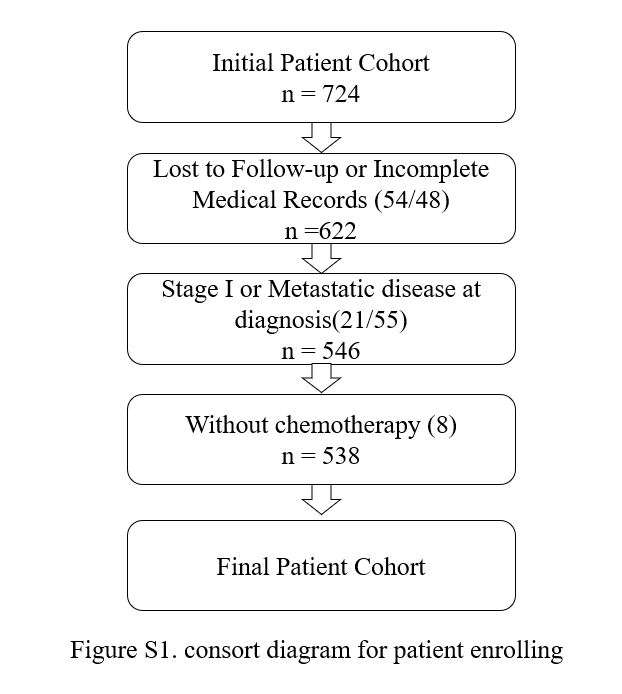

Supplement: Supplementary file 1 — Supplementary Information 1. [file 41598_2022_16216_MOESM1_ESM.png]

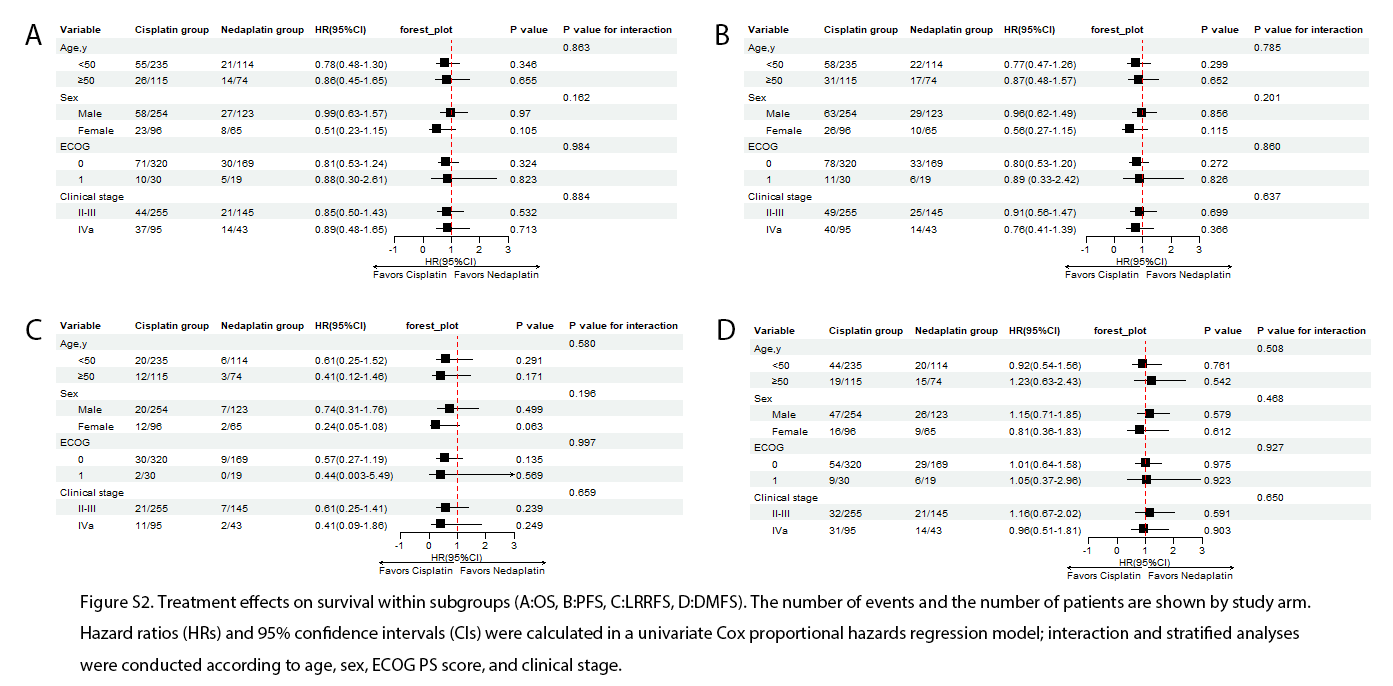

Supplement: Supplementary file 2 — Supplementary Information 2. [file 41598_2022_16216_MOESM2_ESM.tif]
